# Abdominal Adiposity, Not Cardiorespiratory Fitness, Mediates the Exercise-Induced Change in Insulin Sensitivity in Older Adults

**DOI:** 10.1371/journal.pone.0167734

**Published:** 2016-12-09

**Authors:** Gifferd Ko, Lance E. Davidson, Andrea M. Brennan, Miu Lam, Robert Ross

**Affiliations:** 1 School of Kinesiology and Health Studies, Queen’s University, Kingston, ON, Canada; 2 Department of Exercise Sciences, Brigham Young University, Provo, Utah, United States; 3 Department of Epidemiology and Community Health, Queen’s University, Kingston, ON, Canada; 4 Department of Medicine, Division of Endocrinology and Metabolism, Queen’s University, Kingston, ON, Canada; University of Birmingham, UNITED KINGDOM

## Abstract

Abdominal obesity and low cardiorespiratory fitness (CRF) are associated with insulin resistance in older adults. Exercise is associated with improvement in insulin sensitivity. Whether this association is mediated by change in CRF and/or abdominal obesity is unclear. The current study is a secondary analysis of data from a randomized controlled trial in Kingston, Ontario. Sedentary older adults (60–80 years) (N = 80) who completed the exercise (N = 59) or control (N = 21) conditions for 6 months were included. CRF was measured using a treadmill test, adipose tissue (AT) by magnetic resonance imaging, and insulin sensitivity by hyperinsulinemic-euglycemic clamp. Waist circumference (WC) was measured at the iliac crest. Mediation analyses were used to assess whether abdominal AT and/or CRF mediated the exercise-induced change in insulin sensitivity. By comparison to controls, reduction (mean ± SD) was observed for visceral (-0.4 ± 0.4 kg) and abdominal subcutaneous (-0.4 ± 0.4) AT depots, WC (-4.1 ± 3.2 cm) and BMI (-0.9 ± 0.8 kg/m^2^) (p < 0.05). Insulin sensitivity (4.2 ± 5.2 M/I) and CRF (0.2 ± 0.3 L/min) improved in the exercise group (p < 0.05). All AT variables, BMI and WC were mediators of the change in insulin sensitivity (p < 0.05). After adjustment for change in total AT, abdominal AT remained a mediator with an effect ratio of 0.79 (p < 0.05), whereas total AT was not significant when adjusted for abdominal AT (p > 0.05). The effect ratio for change in WC and BMI combined (0.63, p<0.05) was greater than either alone. In conclusion, CRF did not mediate the exercise-induced change in insulin sensitivity in older adults. Abdominal adiposity was a strong mediator independent of change in total adiposity.

## Introduction

Insulin resistance is a strong predictor of morbidity and mortality [1] and aging is associated with a reduction in insulin sensitivity [2]. Aging is also characterized by an increase in abdominal adipose tissue (AT), in particular visceral AT [3], and a decline in cardiorespiratory fitness (CRF) [4], which are both inversely associated with insulin sensitivity [5–7].

It is well established that in older adults, chronic exercise is associated with improvement in insulin sensitivity [8] and CRF [9] as well as a reduction in abdominal obesity [9]. However, whether the change in CRF and/or abdominal obesity mediate the exercise-associated change in insulin sensitivity is unclear. Limited evidence suggests that exercise-induced improvement in insulin sensitivity is associated with reductions in abdominal obesity [7, 10]. Whereas the findings from some short-term intervention studies suggest that changes in CRF are associated with exercise-induced change in insulin sensitivity [7, 11], others do not [10, 12, 13]. Findings from a prior study in middle-aged adults suggest that exercise-induced reduction in insulin resistance was mediated by changes in abdominal obesity, but not CRF [14]. Absent from the literature are data from randomized controlled trials that permit determination of whether changes in abdominal obesity and CRF independently mediate the exercise-induced improvement in insulin sensitivity in older adults.

The purpose of this ancillary study is to identify whether and to what extent CRF and abdominal obesity mediate the association between exercise and insulin sensitivity in older adults. The findings may provide health care practitioners with simple strategies for monitoring the success of a lifestyle intervention designed to improve glucose management.

## Methods

### Participants

136 obese elderly individuals aged 60–80 years participated in the original investigation [9]. Details of the study design and methods in addition to findings from the primary analysis are published elsewhere [9]. This secondary analysis includes 80 subjects in the following three groups: control, aerobic exercise, or a combination of aerobic and resistance exercise. Participants were abdominally obese (≥102 cm waist circumference for men, ≥88 cm for women), and weight stable (±2 kg) for 6 months before study entry. The protocols used in the original investigation were approved by the Queen's University Health Sciences Research Ethics Board. All participants gave informed consent prior to participation.

### Dietary and Exercise Interventions

Participants in the aerobic exercise group were asked to exercise on a treadmill 5 times per week at a moderate-intensity (60–75% VO_2_peak) for 30 minutes each session [9]. The combined exercise group performed 30 minutes of aerobic exercise at a moderate intensity (60–75% VO_2_peak), 3 times per week, plus 3 sessions of resistance exercise per week. Resistance exercises included 1 set of the following 9 exercises, each to volitional fatigue: chest press, shoulder raise, shoulder flexion, leg extension, biceps curl, abdominal crunches, modified push-ups. All participants were instructed by a nutritionist to maintain a healthy, isocaloric diet from baseline and recorded their dietary intake throughout the 6-month intervention [9].

### Anthropometric Measurements

Anthropometric measurements were taken at baseline and at the end of the intervention. Waist circumference (WC) was taken using the mean of 2 measures obtained at the superior edge of the iliac crest.

### Measurement of Total and Regional AT and Skeletal Muscle Mass

Total, abdominal, abdominal subcutaneous, and visceral AT were measured by magnetic resonance imaging using established procedures [15]. Visceral and abdominal subcutaneous AT depots were calculated using 5 images extending from 5 cm below to 15 cm above the L4-5 intervertebral space.

### Measurement of Insulin Sensitivity

Insulin sensitivity was assessed using a 3-hour hyperinsulinemic-euglycemic clamp protocol at baseline and 36–48 hours after the final exercise session [9].

### Cardiorespiratory Fitness Measurement

CRF (measured as oxygen consumption per unit of time [peak VO_2_]) was determined using a maximal treadmill test combined with standard open-circuit spirometry techniques (SensorMedics Corp, Yorba Linda, California) [9]. Briefly, participants walked on the treadmill at a self-selected speed at 0 elevation for 3 minutes, after which the incline was increased every 2 minutes until volitional fatigue.

### Statistical Analysis

The purpose of this investigation was to examine whether changes in CRF and/or abdominal obesity mediate exercise-induced changes in insulin sensitivity in older adults. In the original investigation, both insulin sensitivity and CRF did not change in the resistance training only group. Therefore, of the 136 participants originally randomized, participants were excluded from the final data set if they were in the resistance exercise group (n = 36) and if they did not complete the 6-month intervention. This resulted in a study sample of 80 participants: 1) Exercise (aerobic exercise group and combined exercise group; n = 59) and 2) Control (n = 21).

The group by time interaction was not significant for any of the variables in a repeated measures ANOVA, thus, we collapsed across exercise modality and sex for all statistical procedures. All mediation analyses are adjusted for age and baseline characteristics.

Changes in adiposity, CRF and anthropometric variables in exercise and control groups were compared using independent *t-*tests. Mediation analysis using the product-of-coefficient method [16] was used to delineate the effects of exercise on insulin sensitivity through possible mediators, including CRF, adiposity and anthropometric variables.

Simple mediation was employed, wherein proposed mediators were entered into the mediation model separately to determine each variable’s indirect effects, in addition to the association of exercise and insulin sensitivity after adjusting for the effects of the mediator. Multiple mediation analysis was subsequently used to examine whether mediator variables remained significant after adjusting for the effects of each other. To determine the relative magnitude of the effects, effect ratios were calculated as the ratio of the indirect effect of exercise on insulin sensitivity through the mediator to the total effect of exercise on insulin sensitivity. Statistical procedures were performed using SPSS (IBM SPSS Statistics for Windows Version 21.0. Armonk, NY: IBM Corp.) and a mediation analysis custom dialog (PROCESS) provided by Preacher and Hayes [17].

## Results

Baseline and post-intervention characteristics are shown in Table 1. Significant improvement in insulin sensitivity was observed in the exercise group compared to controls (p < 0.05). Significant reductions were observed for all anthropometric and adiposity variables, and CRF increased in response to exercise compared controls (p < 0.05).

**Table 1:** Participant Baseline Characteristics and Post-intervention Change Scores.

	Exercise Men (N = 26)		Control Men (N = 8)		Exercise Women (N = 33)		Control Women (N = 13)		Exercise Group (N = 59)		Control Group (N = 21)	
	Baseline	Δ	Baseline	Δ	Baseline	Δ	Baseline	Δ	Baseline	Δ	Baseline	Δ
Age, _years_	67.6 ± 5.5		67.7 ± 4.0		67.0 ± 5.8		66.7 ± 4.0		67.3 ± 5.6		67.1 ± 3.9	
BMI, _kg/m_^2^	30.6 ± 3.2	-1.0 ± 0.9	30.0 ± 2.1	0.2 ± 0.3	29.3 ± 3.3	-0.8 ± 0.8	30.3 ± 3.1	0.01 ± 0.5	29.9 ± 3.3	-0.9 ± 0.8	30.2 ± 2.7	0.1 ± 0.5
WC, _cm_	112.3 ± 8.8	-5.0 ± 3.1	111.2 ± 5.2	-0.1 ± 2.4	98.4 ± 9.6	-3.4 ± 3.2	102.4 ± 7.8	-0.2 ± 2.0	104.5 ± 11.5	-4.1 ± 3.2	105.8 ± 8	-0.2 ± 2.1
Body Weight, _kg_	94.9 ± 12.9	-3.0 ± 2.2	91.8 ± 9.9	0.3 ± 0.8	77.9 ± 10.8	-2.1 ± 1.9	82.4 ± 10.1	0.2 ± 1.3	85.4 ± 14.4	-2.5 ± 2.1	86.0 ± 10.8	0.2 ± 1.1
VAT, _kg_	4.6 ± 1.0	-0.6 ± 0.4	3.7 ± 0.7	-0.03 ± 0.2	2.5 ± 0.9	-0.2 ± 0.2	2.7 ± 1.1	0.05 ± 0.2	3.4 ± 1.4	-0.4 ± 0.4	3.1 ± 1.1	0.02 ± 0.2
AbAT, _kg_	9.0 ± 2.5	-1.1 ± 0.7	8.4 ± 1.5	-0.1 ± 0.3	7.7 ± 1.8	-0.6 ± 0.6	8.5 ± 1.9	0.00 ± 0.5	8.2 ± 2.2	-0.8 ± 0.7	8.5 ± 1.7	-0.05 ± 0.4
TAT, _kg_	34.0 ± 8.7	-4.1 ± 2.2	33.1 ± 6.9	-0.7 ± 1.1	35.0 ± 7.2	-2.4 ± 2.0	37.9 ± 7	-0.4 ± 1.4	34.5 ± 7.8	-3.1 ± 2.2	36.0 ± 7.2	-0.5 ± 1.3
ASAT, _kg_	4.4 ± 1.9	-0.5 ± 0.4	4.7 ± 1	-0.1 ± 0.2	5.2 ± 1.4	-0.3 ± 0.4	5.7 ± 1.2	0.04 ± 0.4	4.8 ± 1.7	-0.4 ± 0.4	5.4 ± 1.2	-0.1 ± 0.3
SM, _kg_	30.1 ± 3.6	0.3 ± 1.2	28.9 ± 3.8	0.1 ± 0.8	19.5 ± 3.0	0.4 ± 0.7	20.9 ± 2.8	-0.07 ± 0.7	24.2 ± 6.2	0.3 ± 0.9	23.9 ± 5.1	0.03 ± 0.7
CRF, _L/min_	2.6 ± 0.5	0.3 ± 0.3	2.8 ± 0.4	-0.2 ± 0.3	1.8 ± 0.3	0.2 ± 0.2	1.8 ± 0.3	-0.1 ± 0.2	2.2 ± 0.6	0.2 ± 0.3	2.2 ± 0.6	-0.1 ± 0.3
CRF, L/kg SM/min	0.09 ± 0.02	0.01 ± 0.01	0.10 ± 0.01	-0.01 ± 0.01	0.09 ± 0.02	0.01 ± 0.01	0.08 ± 0.01	0.00 ± 0.01	0.09 ± 0.02	0.01 ± 0.01	0.09 ± 0.01	-0.00 ± 0.01
Insulin Sensitivity, _M/I_	14.6 ± 7.0	4.1 ± 5.1	20.6 ± 8.4	-1.2 ± 5.4	23.0 ± 9.0	4.2 ± 5.3	22.3 ± 8.1	1.2 ± 4.8	19.3 ± 9.2	4.2 ± 5.2	21.7 ± 8.0	0.2 ± 5.0

All data (baseline and post-treatment change scores) given as mean ± standard deviation (SD). All change scores were significantly different from control group (*p <* 0.05). Δ = change from baseline, BMI = body mass index, VAT = visceral adipose tissue, CRF = cardiorespiratory fitness, AbAT = abdominal adipose tissue, TAT = total adipose tissue, ASAT = abdominal subcutaneous adipose tissue, SM = skeletal muscle, WC = waist circumference. M/I, rate of glucose uptake per unit of insulin per kg of muscle mass per minute x 100.

Mediation analysis results are shown in Table 2. In simple mediation, CRF, expressed as L/min or L/kg skeletal muscle/min, was not a mediator of the association between exercise and insulin sensitivity (95% CI: -1.39, 1.36 and -0.32, 5.55, respectively). All AT variables in addition to WC and BMI were mediators of the association between exercise and insulin sensitivity (p < 0.05). After controlling for each adiposity and anthropometric variable separately, the direct effect of exercise on insulin sensitivity was no longer significant (p > 0.05).

**Table 2:** Simple and Multiple Mediation Analysis on the Association between Exercise and Change in Insulin Sensitivity.

Variable	Direct effect of exercise on change in insulin sensitivity (after controlling for the effect of the mediator)		Mediation effect on the association between exercise and change in insulin sensitivity		Effect Ratio (Indirect:Total Effect)
	B(SE)	p-value	Effect(SE)	95% CI	
**Simple Mediation**					
CRF (L/min)	3.53(1.46)	0.02	0.13(0.69)	-1.39,1.36	0.04
CRF (L/kg SM/min)	2.62(1.47)	0.08	0.95(0.82)	-0.34,2.92	0.27
VAT (kg)	2.16(1.32)	0.11	1.62(0.72)*	0.32,3.16	0.43
ASAT (kg)	2.29(1.33)	0.09	1.23(0.51)*	0.43,2.52	0.35
AbAT (kg)	1.68(1.34)	0.21	1.75(0.64)*	0.62,3.13	0.51
TAT (kg)	1.86(1.41)	0.19	1.71(0.67)*	0.54,3.13	0.48
WC (cm)	1.70(1.42)	0.24	1.62(0.74)*	0.36,3.33	0.49
BMI (kg/m^2^)	2.07(1.42)	0.15	1.46(0.64)*	0.29,2.84	0.41
SM (kg)	3.80(1.25)	0.00	-0.21(0.27)	-1.02,0.12	0.06
**Multiple Mediation**					
Total	1.78(1.33)	0.18	1.88(0.76)*	0.51,3.55	0.51
VAT (kg)			0.85(0.79)	-0.54,2.58	0.23
ASAT (kg)			1.03(0.58)*	0.11,2.41	0.28
Total	1.70(1.36)	0.22	2.08(0.78)*	0.66,3.69	0.55
VAT (kg)			0.68(0.98)	-1.16,2.65	0.18
TAT (kg)			1.40(0.92)	-0.29,3.39	0.37
Total	1.91(1.42)	0.18	1.62(0.71)*	0.40,3.22	0.46
ASAT (kg)			0.65(0.84)	-0.92,2.45	0.18
TAT (kg)			0.97(1.17)	-1.15,3.56	0.27
Total	1.54(1.32)	0.25	1.98(0.79)*	0.59,3.71	0.56
AbAT (kg)			2.78(1.43)*	0.41,6.13	0.79
TAT (kg)			-0.80(1.34)	-3.98,1.45	-0.23
Total	1.76(1.39)	0.21	1.99(0.76)*	0.60,3.49	0.53
VAT (kg)			1.17(0.84)	-0.26,3.09	0.31
BMI (kg/m^2^)			0.81(0.80)	-0.68,2.50	0.22
Total	2.10(1.44)	0.15	1.44(0.71)*	0.08,2.91	0.41
ASAT (kg)			0.88(0.72)	-0.49,2.42	0.25
BMI (kg/m^2^)			0.57(0.99)	-1.18,2.77	0.16
Total	1.57(1.45)	0.28	2.09(0.82)*	0.62,3.88	0.57
VAT (kg)			1.18(0.75)	-0.14,2.86	0.32
WC (cm)			0.90(0.85)	-0.58,2.87	0.25
Total	1.27(1.43)	0.38	2.07(0.78)*	0.77,3.93	0.62
ASAT (kg)			1.01(0.57)*	0.11,2.43	0.30
WC (cm)			1.06(0.79)	-0.29,2.90	0.32
Total	1.62(1.43)	0.26	1.88(0.76)*	0.60,3.63	0.54
SM (kg)			-0.11(0.26)	-0.90,0.24	-0.03
TAT (kg)			1.99(0.72)*	0.80,3.67	0.57
Total	1.23(1.47)	0.41	2.09(0.84)*	0.66,4.00	0.63
WC (cm)			1.31(0.96)	-0.36,3.41	0.39
BMI (kg/m^2^)			0.78(0.82)	-0.78,2.48	0.23
Total	2.15(1.50)	0.16	1.36(0.77)	-0.07,2.97	0.39
SM (kg)			-0.07(0.25)	-0.76,0.34	-0.02
BMI (kg/m^2^)			1.43(0.69)*	0.19,2.89	0.41
Total	1.87(1.50)	0.22	1.47(0.86)	-0.08,3.39	0.44
SM (kg)			-0.09(0.28)	-0.86,0.32	-0.02
WC (cm)			1.56(0.77)*	0.27,3.37	0.47
Total	1.51(1.39)	0.28	1.90(0.74)*	0.50,3.43	0.56
SM (kg)			-0.04(0.26)	-0.71,0.42	-0.01
AbAT (kg)			1.94(0.67)*	0.71,3.39	0.57
Total	2.15(1.38)	0.12	1.63(0.83)*	0.08,3.41	0.43
SM (kg)			-0.04(0.22)	-0.67,0.31	-0.01
VAT (kg)			1.67(0.79)*	0.21,3.37	0.44
Total	2.12(1.36)	0.12	1.28(0.64)*	0.24,2.85	0.38
SM (kg)			-0.07(0.25)	-0.79,0.32	-0.02
ASAT (kg)			1.36(0.55)*	0.54,2.86	0.40

All associations were adjusted for age, baseline insulin sensitivity and baseline proposed mediator. Effect ratio = indirect effect / total effect. In the multiple mediation results, “Total” refers to the combined mediation effect of the two listed variables (e.g. combined mediation effects of total AT and abdominal AT). CRF = cardiorespiratory fitness, VAT = visceral adipose tissue, ASAT = abdominal subcutaneous adipose tissue, AbAT = abdominal adipose tissue, TAT = total adipose tissue, WC = waist circumference, BMI = body mass index, SM = skeletal muscle.

* Associations are significant when the 95% confidence interval (CI) does not cross “0”.

**Fig 1:**
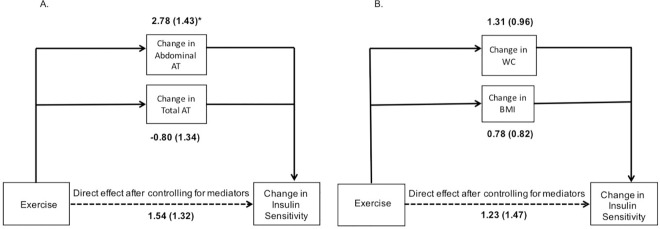
Multiple mediation analysis results for selected variables. AT, adipose tissue; WC, waist circumference; BMI, body mass index. A) Multiple mediation results for abdominal AT and total AT. B) Multiple mediation results for WC and BMI. **Effect (SE)** *Significant association (p<0.05).

Multiple mediation revealed that after controlling for total AT, abdominal AT remained a significant mediator (95% CI: 0.41, 6.13) (Fig 1A) with an effect ratio of 0.79. However, after adjusting for abdominal AT, total AT did not remain a significant mediator (95% CI: -3.98, 1.45). After control for BMI, WC was not a significant mediator (95% CI: -0.36, 3.41); similarly, BMI was not a significant mediator (95% CI: -0.78, 2.48) following control for WC (Fig 1B). However, the effect ratio for changes in WC and BMI combined (0.63) was greater than that observed for either WC (0.49) or BMI (0.41) alone.

Abdominal subcutaneous AT remained a significant mediator after adjusting for visceral AT (95% CI: 0.11, 2.41); however, visceral AT was not significant after adjusting for abdominal subcutaneous AT (95% CI: -0.54, 2.58). Abdominal subcutaneous AT did not remain a significant mediator after adjusting for total AT (95% CI: -0.92, 2.45) or BMI (95% CI: -0.49, 2.42). After adjustment for abdominal subcutaneous AT, total AT (95% CI: -1.15, 3.56) and BMI (95% CI: -1.18, 2.77) did not significantly mediate the association.

## Discussion

The primary finding of this study is that CRF does not mediate the exercise-induced change in insulin sensitivity in older adults whereas change in abdominal AT is a strong mediator independent of total AT. Furthermore, WC and BMI together mediate the association between exercise and insulin sensitivity, suggesting both be measured in clinical settings to assess the efficacy of exercise as a strategy for improving blood glucose management in older adults.

To our knowledge, this is the first study to simultaneously examine the independent effects of exercise-induced change in abdominal obesity and CRF on insulin sensitivity in older adults. Our observation that reduction in abdominal adiposity mediates exercise-induced change in insulin sensitivity substantively extends an earlier report by Stewart et al. wherein an exercise-induced reduction in abdominal adiposity was associated with change in insulin sensitivity in older adults [10]. In that study, however, no attempt was made to determine whether the association between changes in abdominal obesity and insulin sensitivity remained independent of corresponding changes in CRF. We were able to extend these findings through our observation that CRF does not mediate the association.

Our observation that CRF does not mediate the exercise-induced change in insulin sensitivity is consistent with earlier findings in middle-aged adults [14]. Prior small-sample intervention studies that observe a significant association between exercise-induced change in CRF and insulin sensitivity do not adjust for covariates, precluding the conclusion that change in CRF, independent of change in adiposity, is associated with exercise-induced change in insulin sensitivity [7, 11]. Our observations are consistent with others [10, 12, 13], suggesting that CRF is a characteristic that may improve consequent to increases in physical activity, but does not provide a mechanism by which exercise improves insulin sensitivity. Since improvement in CRF is driven by improvement in cardiac output [18]. it follows that change in insulin sensitivity, a peripheral adaptation in skeletal muscle glucose uptake [19], is not associated with changes in CRF. This finding does not negate the importance of measuring CRF in practice as it is established that CRF is an independent predictor of morbidity and mortality in older adults [20]; however, it suggests that the change in CRF that occurs in response to exercise does not inform clinicians regarding the individual’s ability to manage blood glucose in the short term.

That reduction in abdominal adiposity mediates the association between exercise and insulin sensitivity independent of total adiposity is consistent with a large body of cross-sectional evidence confirming a strong independent association between abdominal obesity and insulin sensitivity [7, 21]. The putative mechanisms that link abdominal AT with cardiometabolic risk factors including insulin resistance continue to be the source of intense investigation [22]. Whether the mechanisms be of a substrate [23] and/or cytokine origin [22, 24], the importance of the exercise-induced decrease in abdominal adiposity is underscored by the fact that the absolute reduction in abdominal AT in our study was approximately one-third that of total adiposity (Table 1). Thus the findings from this report and others [7, 10] reinforce the importance of reducing abdominal obesity for improving the management of insulin sensitivity in older adults.

Our finding that change in both WC and BMI is a stronger predictor of the exercise-induced reduction in insulin resistance compared to either WC or BMI alone differs from previous observations in middle-aged adults wherein WC, independent of BMI, mediates exercise-induced change in insulin sensitivity [14]. However, it is consistent with cross-sectional observations suggesting that combining WC with BMI measurement predicts cardiometabolic risk better than either alone [25]. Taken together, these findings suggest that both WC and BMI are simple anthropometric tools that should be measured in clinical practice to both identify high risk phenotypes and assess the efficacy of strategies designed to improve glucose management in older adults.

Our observation that reductions in abdominal subcutaneous AT mediate the exercise-induced reduction in insulin sensitivity independent of change in visceral AT whereas the reverse is not true was an unexpected finding with important implications. We [26] and others [6, 27] have previously observed that changes in visceral AT are associated with change in insulin sensitivity independent of abdominal subcutaneous AT. However, others report a strong and independent association between abdominal subcutaneous AT and insulin resistance [28–30]. Taken together, these findings reinforce the importance of both abdominal subcutaneous AT and visceral AT in relation to insulin sensitivity in older adults and suggests that strategies designed to improve insulin sensitivity in the elderly are optimized when both depots are mobilized in response to an exercise-induced reduction in BMI and/or waist circumference.

Limitations to our study include the homogeneity of the participants which narrows the generalizability of our findings. However, given that the worldwide prevalence of aging and related obesity is already high and increasing, our findings are relevant for a large segment of the older adult population with the high-risk form of obesity. Although our analysis included criterion measures of AT distribution as potential mediators, other body composition variables including liver fat [31] and intramuscular lipid [32] known to be associated with insulin sensitivity were not measured. Additionally, participants were not monitored for potential activity outside of the intervention. The strengths of our study include rigorously controlled and supervised exercise protocols and the use of criterion methods to measure insulin sensitivity and whole-body adiposity. The use of mediation analysis allowed us to separate the total effect of exercise on insulin sensitivity into the direct and indirect (via the mediator) effects. This type of analysis addresses the limitations of commonly employed traditional regression analysis, which fails to identify whether or not a covariate is a true mediator, described as a variable associated with both the exposure (exercise) and the outcome (insulin sensitivity).

In summary, our findings suggest that change in abdominal obesity, not CRF, mediate the exercise-induced improvement in insulin sensitivity in older adults. Further, WC and BMI combined are simple tools that mediate the exercise-induced improvement in insulin sensitivity better than either measure alone. Health care providers are encouraged to obtain these anthropometric measures in order to determine the efficacy of exercise combined with a healthful diet as a strategy to improve insulin sensitivity in older adults.

## Supporting Information

S1 File Minimal Dataset to Support Study Findings. (XLSX)
